# Neurocognitive Assessment of Mathematics-Related Capacities in Neurosurgical Patients

**DOI:** 10.3390/brainsci14010069

**Published:** 2024-01-10

**Authors:** Elisa Castaldi, Camilla Bonaudo, Giuseppe Maduli, Giovanni Anobile, Agnese Pedone, Federico Capelli, Roberto Arrighi, Alessandro Della Puppa

**Affiliations:** 1 Department of Neuroscience, Psychology, Pharmacology and Child Health, University of Florence, 50135 Florence, Italygiovanni.anobile@unifi.it (G.A.); roberto.arrighi@unifi.it (R.A.); 2 Neurosurgery, Department of Neuroscience, Psychology, Pharmacology and Child Health, University of Florence, University Hospital of Careggi, 50134 Florence, Italy; camilla.bonaudo@unifi.it (C.B.); agnese.pedone92@gmail.com (A.P.); federicocapelli27@gmail.com (F.C.); alessandro.dellapuppa@unifi.it (A.D.P.)

**Keywords:** numerosity perception, non-symbolic geometrical reasoning, neuropsychological assessment, neurosurgery, oncology

## Abstract

A precise neuropsychological assessment is of the utmost importance for neurosurgical patients undergoing the surgical excision of cerebral lesions. The assessment of mathematical abilities is usually limited to arithmetical operations while other fundamental visuo-spatial aspects closely linked to mathematics proficiency, such as the perception of numerical quantities and geometrical reasoning, are completely neglected. We evaluated these abilities with two objective and reproducible psychophysical tests, measuring numerosity perception and non-symbolic geometry, respectively. We tested sixteen neuro-oncological patients before the operation and six after the operation with classical neuropsychological tests and with two psychophysical tests. The scores of the classical neuropsychological tests were very heterogeneous, possibly due to the distinct location and histology of the tumors that might have spared (or not) brain areas subserving these abilities or allowed for plastic reorganization. Performance in the two non-symbolic tests reflected, on average, the presumed functional role of the lesioned areas, with participants with parietal and frontal lesions performing worse on these tests than patients with occipital and temporal lesions. Single-case analyses not only revealed some interesting exceptions to the group-level results (e.g., patients with parietal lesions performing well in the numerosity test), but also indicated that performance in the two tests was independent of non-verbal reasoning and visuo-spatial working memory. Our results highlight the importance of assessing non-symbolic numerical and geometrical abilities to complement typical neuropsychological batteries. However, they also suggest an avoidance of reliance on an excessively rigid localizationist approach when evaluating the neuropsychological profile of oncological patients.

## 1. Introduction

The neurocognitive assessment of neurosurgical patients is acquiring more and more importance in clinical practice. The principle of onco-functional balance is the guiding light for neurosurgeons, and many methods may be used to achieve maximal safe resection, achieving progression-free survival and granting a high quality of life [1,2,3,4,5,6,7]. Neuropsychological evaluation is normally performed both in the presurgical setting to develop preoperative plans as well as after surgery to evaluate post-operative outcomes. Before surgery, neuropsychological tests evaluating various cognitive functions are administered during navigated transcranial magnetic stimulation (nTMS) to outline eloquent and non-eloquent areas and relate them to both the cerebral lesion and the white matter fibers reconstructed by means of MRI scanning [8,9,10,11]. In cases where patients can undergo surgery whilst awake, neuropsychological tests are performed during intraoperative mapping as well while direct cortical stimulation is delivered in cortical or subcortical positive sites (previously identified by nTMS). Post-operative assessment is then performed to evaluate surgical outcomes or the effects of rehabilitation programs.

Despite its crucial role in all phases of neurosurgical procedures, neurocognitive assessment is generally limited to very few cognitive functions, which are often just evaluated coarsely. For example, language functions are simply tested by asking patients to name figures; visuospatial abilities are assessed by means of line bisection or cancellation tasks; social abilities are monitored by asking patients to identify emotions in photos; and motor functions are assessed by inviting patients to perform simple movements with their wrist [12]. Therefore, the modern neurosurgical approach, either in the oncological or vascular field, urges the exploration of more neurocognitive functions that are not deeply and precisely investigated by the currently available tests, following the direction of ‘tailored-connectome-based-surgery’ [13], with as much respect to significant functions as possible.

Among all cognitive functions, the assessment of mathematical abilities, when performed, is usually limited to simple arithmetical operations [14,15,16], while other fundamental non-verbal, visuo-spatial aspects of mathematics, such as the perception of numerical non-symbolic quantities (numerosity) and geometrical reasoning, are not evaluated at all. Many daily tasks, such as organizational actions, require the processing of quantitative information (e.g., choosing the correct quantity of ingredients while cooking, planning groceries based on the needs of the household, and handling money and measurements with a reasonable understanding of their order of magnitudes) and are challenging to envision without a proficient perception of non-symbolic quantities. Moreover, undertaking activities that necessitate the ability to visualize, plan, and execute spatial designs while comprehending and manipulating spatial relationships (e.g., anticipating or remembering the trajectory of a moving target, path selection in navigation, and engaging in creative endeavors or construction activities) would be challenging to carry out effectively without the support of non-verbal geometrical reasoning. In addition to their likely relevance to daily living, a large body of evidence suggests that these abilities are closely linked to symbolic mathematics proficiency. The precision of numerosity discrimination in children predicts their current or future mathematical proficiency [17,18,19,20,21], suggesting that the perception of non-symbolic numerical quantities might constitute a foundational start-up tool for symbolic number representation during development [22]. The relationship between the perception of non-symbolic numerical quantities and mathematical proficiency is observed throughout a person’s lifespan [23], and training the non-symbolic numerosity system improves symbolic addition and subtraction not only in children, but also in adults [24,25]. Both children and adults with developmental dyscalculia, a specific disability in learning formal mathematics, are less precise when asked to determine which out of two ensembles of dots is more numerous [26,27,28,29,30]. Although not exempt from criticism, in light of this evidence, an influential theory suggests that the perception of non-symbolic quantities is fundamentally linked to mathematics skills [31]. This theory also proposes that the ability to perceive non-symbolic quantities crucially relies on the functionality of the intraparietal sulcus [32]. Neuroimaging studies have repeatedly demonstrated that in neurotypical adults, both the ability to perceive or manipulate non-symbolic numerical quantities, as well as the ability to solve simple arithmetic operations, require a wide fronto-parietal network [33,34,35], and that functional and anatomical alterations to this network can hamper both of these abilities [36,37,38]. The involvement of the parietal cortex in calculation processing has been also confirmed by direct electrical stimulation studies during awake surgery [14,15,16].

Together with arithmetics, formal geometry is another important branch of mathematics likely rooted in a non-symbolic system supporting geometrical intuition. Human infants are equipped with some rudimentary geometrical knowledge and are sensitive to shape, angular size, object distance from landmarks, and relative length [39,40,41]. Even adults with no formal instruction in geometry, such as the indigenous of the Amazonian Munduruku population, are sensitive to geometrical cues [42]. Recently, non-symbolic geometric reasoning has been investigated with a paradigm in which a cursor is sequentially flashed across eight possible positions around an octagon and, after observing a sequence of the first five locations, participants are asked to predict the future cursor locations [43]. The sequences follow different geometrical paths of increasing complexity. In contrast to other tests used to investigate non-symbolic geometry, such as those involving the identification of a disparate geometrical shape out of multiple alternatives [42,44], the paradigm devised by Amalric et al. [43] requires individuals to integrate information across time and involve a certain level of syntactic representation, thereby measuring a sort of “non-verbal language of thought”. Indeed, sequences are created from geometrical primitives that are then applied in a recursive manner, similarly to what occurs in verbal language. Despite the apparent complexity of this task, both pre-schoolers and indigenous Munduruku people can quickly perceive and predict these geometrical sequences notwithstanding their lack of education in formal geometry [43]. A recent study found that perception of these geometric sequences predicts formal geometric abilities in primary school children [45], echoing the link between symbolic and non-symbolic systems suggested for arithmetics and numerosity perception. An fMRI study identified the neural substrate supporting perceptions of non-symbolic geometrical sequences [46]. Specifically, Wang et al. [46] found that sequence complexity was coded by a bilateral region in the dorsal inferior frontal gyrus (dorsal to the language-related areas in BA44), a region that is also activated by abstract mathematical thinking [47].

The current study aimed to achieve two primary objectives. First, we aimed to expand the range of functions evaluated in neurosurgical patients by testing both the perceptions of non-symbolic numerosity and geometry, abilities of key importance in many everyday life situations. Our goal was to gain preliminary insights into the extent to which performance in the two psychophysical tests is independent from performance in other classical neuropsychological tests, thereby enriching and complementing them. Second, we aimed to evaluate whether performance in the two psychophysical and classical neuropsychological tests can reliably serve as a predictive indicator of lesion locations at the individual patient level. In the light of the reviewed neuroimaging literature, we expected the two non-symbolic tests to be sensitive to the degree to which the frontal and parietal regions function. However, since most of the neurosurgical patients undergoing surgical excision are affected by tumors, we directed particular attention to individuals’ variability in cognitive functioning in relation to the lesion site. Indeed, in contrast to patients affected by acute events, such as strokes, tumors might develop gradually over time, allowing for a different degree of plastic reorganization. Moreover, tumors might infiltrate and interrupt the functionality of connecting fibers, which might result in the dysfunction of regions that are distant from the lesion. We anticipate that while the two psychophysical tests provide additional information that would have not been otherwise captured by the classical neuropsychological tests, some caution should be taken when evaluating oncological patients as their neuropsychological profile might not closely follow the one expected based on lesion location.

## 2. Materials and Methods

### 2.1. Participants

Data collection was carried out between January 2021 and November 2022. In this period, more than 400 neuro-oncological patients were operated on at the Neurosurgical Department of the University Hospital of Florence, and among these we enrolled only patients with small lesions (<4 cm, although some lesions had a major volume in some axes at the preoperative MRI scan) located in precise intralobular anatomical regions. This selection aimed to study the relationship between the loss (or preservation) of specific cognitive and perceptual abilities and the lesion’s location, which could have perturbed (or spared) the functionality of key areas thought to support the functions under investigation, at least as suggested by neuroimaging studies in neurotypicals. Therefore, the inclusion criterion was having a small lesion located in specific intralobular regions and an age > 18 years. Exclusion criteria were an age < 18 years old, an inability to provide informed consent to the study, and pregnancy. As a result of this selection, 16 patients were enrolled in the study (5 females; 54 ± 13 years old; 11 ± 3 years of schooling, all right-handed). None of our patients disclosed a previous psychiatric diagnosis, and none had a history of taking psychotropic medications, as revealed during the medical history screening. None of our patients had received a diagnosis of executive dysfunction in the past. Although these affective or executive difficulties were not specifically tested in the current study, all participants exhibited a cooperative and engaged demeanor both before and after the surgery. We considered patients with various histological lesions and patterns of growth (8 glioblastoma, 1 artero-venous malformation, 1 choriocarcinoma, 1 renal tumor’s metastasis, 1 pulmonary tumor’s metastasis, 2 cavernous angioma, 1 oligodendroglioma WHO grade II, and 1 astrocytoma), located in different sites of the brain (occipital = 1, temporal = 3; parietal = 5, frontal = 7; individual patients’ details are reported in Table 1). We enrolled patients with precise intralobular anatomical regions, except for patient S16, whose lesion, despite being relatively small, covered both frontal and temporal areas. The frontal lesion in patient S16 was located in the areas presumably involved in non-symbolic geometry perception according to the literature [46]; therefore, this patient was included in the group of patients with frontal lesions. Participants with temporal and occipital lesions (with no visual deficits) served as controls, as the non-symbolic geometry and numerosity perception tests were mainly designed to evaluate the functionality of the front-parietal network. Six patients were tested both before and after the surgery (at least seven days after operation). All patients had normal or corrected-to-normal vision both before and after surgery.

The experimental protocol was approved by the local ethics committees in accordance with the Declaration of Helsinki. All patients provided written informed consent for all medical evaluations and treatments. The study was conducted in accordance with the Declaration of Helsinki and approved by the ethics committee CEAVC (Comitato Etico Area Vasta Centro), a section of the Regional Ethics Committee of the Region of Tuscany (DGRT 418/2013, protocol code 17003_oss, date of approval: 21 July 2020).

### 2.2. General Surgical and Experimental Procedures

Surgical excision was performed by the same Senior Neurosurgeon (A.D.P.), with gross total resection (GTR) achieved in all cases (i.e., >95% of lesion macroscopically removed). GTR was achieved in all cases and observed with a postoperative CT scan, performed for all patients. In cases of patients with a suspected diagnosis of high-grade glioma, 5-Aminolevulinic acid (5-ALA) was used to improve the extent of resection and check the tumor margins. Moreover, in cases of brain metastases, sodium fluorescein was administered to better distinguish pathological from healthy tissue. General anesthesia was administered in 12 cases, (the 4 awake procedures being S5, S8, S9, and S12), conducted with the anesthesiologic protocol of ‘asleep–awake–asleep’. The regular duration of the operation was between 3 h and 6 h, according to the size and location of the tumors, the asleep/awake procedures chosen, and considering either anesthesiologic or surgical times. Eleven patients received a preoperative antiepileptic treatment (eight with Levetiracetam, two with Lacosamide, and one with a double therapy of Lacosamide and Carbamazepine; see Supplementary Table S1). Patients’ therapy depended on the type of histological diagnosis. For patients with glioblastoma, the standardized Stupp Protocol was administered [48]. For the other patients, dexamethasone and antiepileptic drugs were normally administered during the 30 days after surgery, and then therapeutic variations were decided according to histological diagnosis, clinical conditions, and response to other treatment. For oncological patients with brain metastases, after interdisciplinary team discussion with members of the tumor board, oncological therapies were adapted to each patient, based on the primary tumor and source of brain lesion. The duration of the disease, which is difficult to estimate precisely, differed based on the histological nature. Patients with vascular lesions (AVM and cavernomas) were epileptic, and MRI scans were performed to confirm the presence of cerebral lesions, which justified seizures because of their location and irritation on the cerebral cortex. Surgical treatment was considered after refractory antiepileptic therapy (average of 8–12 months after clinical–radiological diagnosis). Patients with low-grade or high-grade gliomas were admitted to our department after imaging studies and were operated on immediately after diagnosis (LGG with an average of 60 days after diagnosis; HGG with an average of 30 days after diagnosis). Finally, patients with brain metastases had different medical stories, based on the primitive tumor location. Two of them (S9 and S11) had already undergone a cycle of medical treatment (chemo-radiotherapy), and they were followed up clinically and radiologically after surgery. One patient (S11) had a second smaller lesion at a different location, whose treatment was performed with gamma knife (radiosurgery) after surgical excision of the most clinically impacted one.

Between 24 h and 72 h before the operation, patients were tested with a series of psychophysical and neuropsychological tests for about two and a half hours. Six patients underwent the same tests after surgery, on average 5 ± 3 days after the operation. The participants’ perceptions of non-symbolic geometry and numerosity were measured via two psychophysical tests, administered in a pseudorandom order followed by a standardized battery of neuropsychological tests. Visuospatial and verbal working memories were measured with the Corsi block tapping test and the Digit Span subtest from the WAIS-IV battery, respectively; verbal and non-verbal reasoning were indexed by performance to Similarities and Matrix Reasoning subtests from the WAIS-IV battery, respectively. Patients were tested by the same operator to reduce all possible examiner biases.

### 2.3. Psychophysical Tests

Stimuli used in the psychophysical tests were created with MATLAB R2021a using PsychTool-box routines [49] and presented on a 12.3″ touchscreen tablet (Microsoft Surface Pro, Microsoft Corporation, Redmond, WA, USA), with 2736 × 1824 resolution and a refresh rate of 60 Hz. Participants viewed the stimuli from an approximate 60 cm distance.

#### 2.3.1. Non-Symbolic Numerosity Perception Test

**Figure 1:**
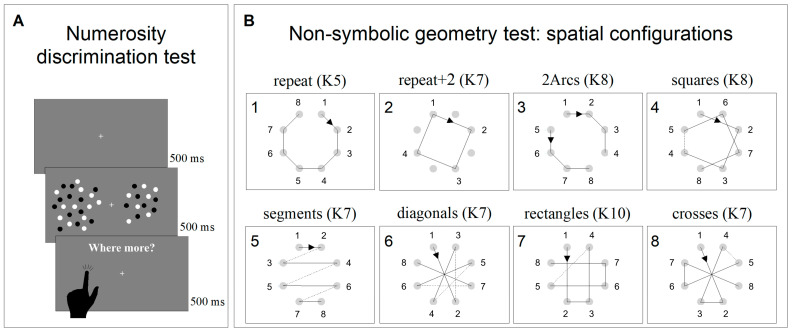
Psychophysical non-symbolic tests. (**A**) Representation and timeline of the non-symbolic numerosity discrimination test. (**B**) Representation of the eight geometrical sequences tested in the non-symbolic geometry test. The numbers near the small circles represent the order followed by the black square. The numbers on the upper left corner of the boxes represent the presentation order, and sequence complexity (K) is specified for each sequence.

Numerosity discrimination thresholds were obtained using a discrimination test (two alternative forced choice—2AFC). The stimuli comprised two arrays of dots briefly (500 ms) presented on either side of a central fixation point. After the stimuli disappeared, individuals were asked to indicate which patch was more numerous by touching the corresponding side of the screen (Figure 1A). The numerosity of the probe was 24, while the test adaptively changed between 3 and 600, according to the QUEST algorithm, perturbed with Gaussian noise with a standard deviation of 0.15 log units. All participants performed two sessions of 90 trials each (180 trials in total for each participant). Dots were 0.2° in diameter, presented at 90% contrast on a grey background of 40 cd/m^2^. Within each patch, dots were half white and half black, so that luminance levels did not vary with numerosity. Dots were constrained to fall within a virtual circle of 5° × 5°, centered at 7° eccentricity.

#### 2.3.2. Non-Symbolic Geometry Test

The non-symbolic geometry perception was measured with an adapted version of the paradigm devised by Amalric et al. [43]. At the beginning of each trial, eight grey dots equally spaced around a virtual circle (9° diameter) appeared and remained onscreen for the entire length of the experiment. Soon after, a black square (0.8° × 0.8°) appeared and was sequentially flashed at (some of) the eight locations marked by the grey dots following a specific virtual geometrical sequence. Each black square was presented for 1000 ms and separated by the following one by a fixed inter-stimulus interval of 300 ms. Participants were then asked to complete the geometrical sequence by sequentially tapping on the dots corresponding to the locations in which they thought the square would have appeared next. If they tapped on the wrong dot, a short sound was played, and the black square started moving again following the already-shown sequence plus the last location in which the mistake occurred. This procedure was implemented until the whole sequence (comprising eight locations) was completed.

Each sequence was tested twice in separate blocks (1st and 2nd runs hereafter), which aimed to measure sequence anticipation and memory, respectively. During the 1st run, a black square was flashed on five successive locations and participants were asked to complete the sequence by tapping on the next three locations. After the 1st run, the screen turned grey, and the 2nd run was started by the experimenter pressing the space bar on the keyboard. During the 2nd run, the same sequence was tested, but the square moved only across the first three locations and participants had to complete the sequence by tapping on the five following steps. Again, after the 2nd run, the screen turned grey, and the following trial, testing a different geometrical sequence, was started by the experimenter by pressing the space bar. Eight different sequences were tested (Figure 1B). Each sequence was associated with a degree of complexity (K) that reflected geometrical regularity. Higher K values indicated higher complexity and less regularity (for a complete description of how to calculate K, see Amalric et al. [43]). In contrast to the original study, in which the eight sequences were randomly presented, in the current study, we presented them in the same blocked order of increasing difficulty. Sequence difficulties were estimated based on the average performance of 5-year-old children [43]. The order of the sequences was: repeat (K5), repeat + 2 (K7), 2arcs (K8), squares (K8), segments (K7), diagonals (K7), rectangles (K10), and crosses (K7). Sequences were presented in blocked order to reduce the inter-subject variability induced by hysteresis (the influence of the difficulty of the previous N-1 session on the following one). In Amalric et al.’s [43] study, the “4-segments” sequence was presented 4 times, in order to test all 4 axial symmetries; however, in the current study, it was presented only once.

### 2.4. Data Analysis

Scores obtained in the neuropsychological tests were corrected according to the standardized normative tables.

No norms exist for the two non-symbolic tests; however, the non-symbolic geometry test has previously been administered to neurotypical adults [43] and children [45], and we referred to these values to define the normative range. To measure the neurotypical performance on the other non-symbolic test (numerosity discrimination test), we tested sixteen additional age-matched neurotypical participants (12 females, 4 males; 51 ± 14 years old, minimum age: 28 years old, maximum age: 73 years old; 15 ± 3 years of schooling).

For the numerosity discrimination test, we plotted the proportion of “test more numerous” responses against the numerosity of the probe (on log axis) and fitted the data with a cumulative Gaussian error function. For each participant, we quantified the goodness of fit by measuring the coefficient of determination (R-squared). R-squared values lower than 0.5 were obtained when patients were not able to perform the task and provided random responses that could not be reasonably fitted. In all other cases, we defined as just notable difference (JND) the difference in numerosity between the 50% and 75% points on the psychometric function. JNDs were then used to calculate a dimensionless psychophysical index for discrimination thresholds (Coefficient of Variation; CV = JND/PSE). Higher discrimination thresholds indicate lower precision. In the case of unrealistically high coefficients of variations (calculated from best fitting curves with R-square lower than 0.5), these CV values were discarded from subsequent analyses. Numerosity discrimination thresholds measured on a group of healthy age-matched participants were used to calculate confidence intervals.

For the non-symbolic geometry test, for each participant, we calculated the proportion of correct responses for each sequence and averaged these values across the eight sequences. These values were compared with those previously measured in healthy adults [43] and primary school children [45], which we used to calculate confidence intervals.

The average performance in the non-symbolic geometry test was compared across groups of patients with different lesions using the 10,000-iteration Bootstrap sign test, adopting sample-with-replacement [50]. On each iteration, we computed the group accuracy and the accuracy difference between groups. In matching the different groups, we calculated as p the proportion of times the difference between two groups was higher than 0 on the total number of iterations. The accuracies of patients with parietal and frontal lesions were compared with those with occipital an d temporal lesions (used as a control group), but also between patients with parietal lesion relative to those with frontal lesions.

Data were analyzed using Matlab R2021a (https://it.mathworks.com, accessed on 1 January 2021) and Jasp (version 0.8.6, The JASP Team 2020, https://jasp-stats.org, accessed on 1 January 2020). Graphs were created with OriginPro (10.0) 2023 (https://www.originlab.com/, accessed on 1 January 2023).

## 3. Results

### 3.1. Neuropsychological Assessment

The neuropsychological assessment was completed in most of the participants, except for one patient with a parietal lesion and one with a frontal lesion who could not complete tests one and two, respectively, due to lack of compliance at the end of the testing session. Despite the lesions being quite specific, cognitive profiles were highly heterogeneous even across patients with lesions circumscribed to the same lobe. Based on the scores in the classical neuropsychological tests, it was therefore not possible to directly predict the lesion sites. Moreover, the surgery did not appear to have a clear impact on patients’ cognitive abilities.

### 3.2. Non-Symbolic Numerosity and Non-Symbolic Geometry Tests

All participants were tested with both the non-symbolic numerosity and geometry tests; six of them were also evaluated with these tests after the surgery.

**Figure 2:**
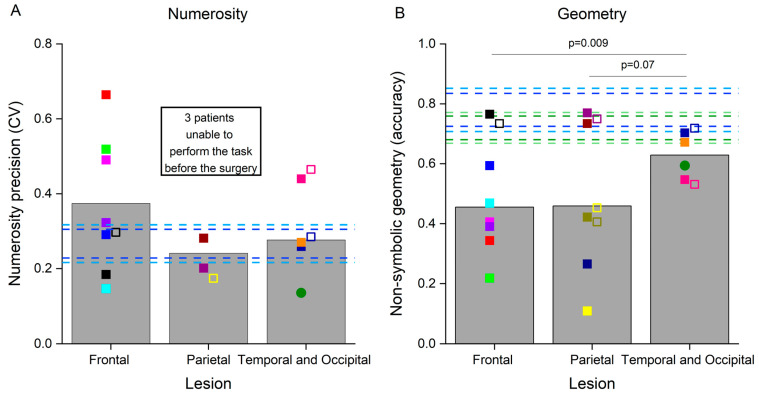
Performance in the non-symbolic tests. Bars represent the average results in the non-symbolic numerosity discrimination (**A**) and non-symbolic geometry (**B**) tests in the three groups of patients before the surgery. Colored symbols represent the numerosity discrimination precision (**A**) and the accuracy of responses in the non-symbolic geometry test (**B**) in individual patients before (filled symbols) and after (open symbols) the surgery. The circle represents the performance of the patient with an occipital lesion. Dashed horizontal bars represent the 95th and 99th percentiles of the neurotypical scores measured in adults (dark and light blue lines, respectively) and in primary school children (dark and light green lines, respectively). Individual participants are color-coded.

We quantified the precision of numerosity discrimination by means of the coefficient of variation, indexing numerosity discrimination thresholds, with higher values corresponding to lower precision (Table 1 and Figure 2A). Numerosity discrimination thresholds exceeded the 99th percentile of the neurotypicals’ value in three patients with frontal lesions, while they were within the neurotypical range in the other four. After the surgery, the coefficient of variation measured in one frontal patient with previously very good numerosity discrimination abilities increased (indicating a reduced precision), yet remained within the neurotypical range. Three patients with parietal lesions were strongly impaired in this test and, before the surgery, were already providing random responses which prevented the fitting routine to reasonably interpolate the data (R^2^ lower than 0.5, marked as n.a. in Table 1). Numerosity discrimination precision in the other two patients with parietal lesions was within the neurotypical range. Notably, after the surgery, one of the patients with a parietal lesion, who was previously unable to perform the test, demonstrated very good numerosity discrimination abilities. Numerosity discrimination measured in three patients with occipital and temporal lesions was within the neurotypical values, and impaired in one patient with a temporal lesion. Numerosity discrimination thresholds remained largely constant in these patients after the surgery. Mean group differences in CVs were not statistically compared across groups, because CVs could only be reliably estimated in two patients with parietal lesions. However, the fact that the other three patients with parietal lesions were not even able to perform the test strongly suggests the crucial role of the parietal cortex in solving this task, in line with multiple evidence described in the literature.

Participants were also tested with a non-symbolic geometry test (Table 1 and Figure 2B). Six patients with frontal lesions were strongly impaired in this test, while one patient with a frontal lesion performed it with accuracy levels within the neurotypical range both before and after surgery. Three patients with parietal lesions were also very inaccurate in this test, and the performance did not normalize in those tested after the surgery. The other two patients with parietal lesions yielded accuracies within the neurotypical range, and the one tested after surgery was still very accurate. Finally, accuracy in this test was also slightly lower than the neurotypical range in two patients with occipital and temporal lesions, while two patients with temporal lesions performed within the neurotypical range for adults or children, even if close to the borderline value of the 99th percentile. Accuracy in the geometry test remained quite constant after the surgery in these patients. Variability between patients with temporal and occipital lesions was much smaller compared with that present in the other two groups and similar to the variability present in the neurotypical population. We quantified between-group differences in accuracy by means of bootstrap sign tests, which revealed that patients with temporal and occipital lesions performed the non-symbolic geometry test significantly better than those with frontal lesions (*p* = 0.009). This difference was marginally significant when comparing patients with temporal and occipital lesions with those with parietal lesions (*p* = 0.07), and not significant when comparing patients with frontal lesions against those with parietal lesions (*p* = 0.5).

We also analyzed patients’ performance in the first (Figure S1A) and the second (Figure S1B) runs of the non-symbolic geometry test separately, as these are thought to rely more on geometrical intuition and memory, respectively. However, the within-participant performance values were, in most cases, quite similar across runs and not clearly impacted by the surgery. Importantly, individual performance in the non-symbolic geometry test, both across and within runs, did not clearly reflect the scores in the classical neuropsychological tests. This observation is discussed in the following paragraph.

### 3.3. Single Cases

**Figure 3:**
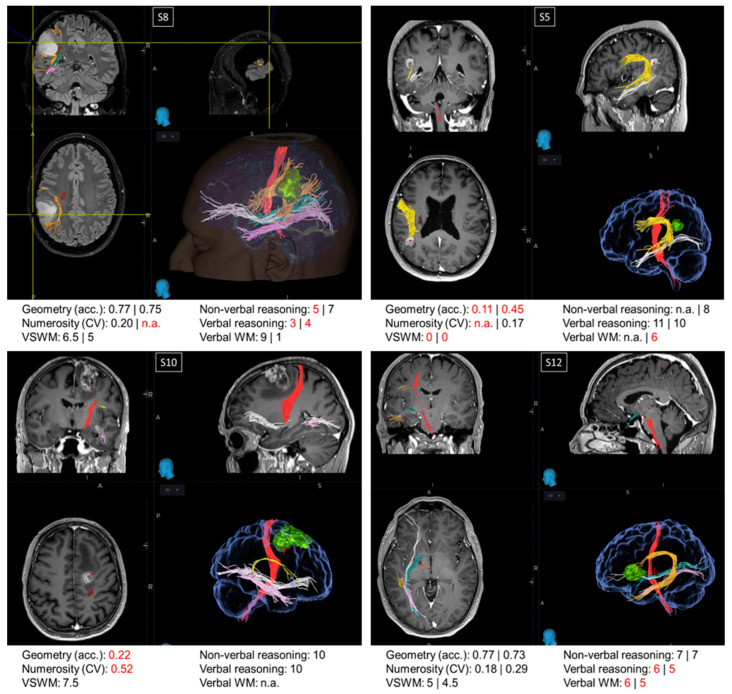
Single cases. Images from the NeuroNavigation system, Medtronic (reading left image_left side/right image_right side): MRI images displaying the lesions and scores in neuropsychological and psychophysical tests in patients S8, S5, S10, and S12. When two values are present, the first refers to the score before the surgery and the second refers to the score obtained after the surgery. Scores below the normative value are marked in red. Major fibers obtained by MRI DTI before the surgery are color-coded as follows: red: corticospinal tract; orange/yellow: arcuate fasciculus; green: optic radiations; pink: inferior longitudinal fasciculus (ILF); white: inferior fronto-occipital fasciculus (IFOF).

Oncological lesions often compromise a large portion of the patients’ brain, making the relation between the loss (or preservation) of a certain function and the functionality of a specific region hard to trace. For this reason, from a pool of more than 400 neuro-oncological patients operated on at the Neurosurgical Department of the University Hospital of Florence over almost two years, we selected 16 patients with small lesions located in precise intralobular anatomical regions. Although this selection resulted in a relatively restricted sample size, it offered the opportunity to evaluate single cases, which is often standard practice in neuropsychology. Many authors consider single case studies as a powerful neuropsychological technique [51] as they offer the opportunity for in-depth testing of the brain lesions of one or a few patients alongside a precise assessment of the cognitive abilities impaired as a consequence of the brain circuits’ disruption. Indeed, many of the most crucial discoveries in the field of neuropsychology have been gathered via single case studies through the description of single or double dissociations of the cognitive functions. In this section, we describe the cognitive profile of four patients (Figure 3). Examination of the first three single cases (S5, S8, and S10) suggested that the non-symbolic geometry test measures abilities additional to those measured by non-verbal reasoning and non-verbal working memory tests. The last single case (S12) is discussed because it was particularly informative for the localization of the regions crucial to perform the non-symbolic geometry test accurately.

Patients S5, S8, and S10. Patient S8 and patient S5 exhibited a double dissociation between their scores in the non-symbolic geometry and non-verbal reasoning tests. Before surgery, patient S8, with a parietal lesion, performed the non-symbolic geometry test with very high accuracy; however, their performance in the non-verbal reasoning test was below the normative value. On the other hand, patient S5, also exhibiting a parietal lesion, was very inaccurate in the non-symbolic geometry test both before and after surgery, despite scoring within the normative range in the non-verbal reasoning test after the surgery (scores before the surgery not available). While the comparison between these two patients suggests a dissociation between non-symbolic geometry and non-verbal reasoning, performance on the non-symbolic geometry test in these patients could potentially be fully explained by the functionality of their visuo-spatial working memory, which was spared in S8 and impaired in S5, both before and after the surgery. This means that, if these two patients simply relied on their visuo-spatial working memory, they would have likely scored the same in the non-symbolic geometry test, without necessarily having grasped that the square was moving following a geometrical trajectory (rather than randomly). However, this explanation does not hold when considering patient S10, who had a frontal lesion. Similarly to patient S5, S10 performed poorly on the non-symbolic geometry test despite normal non-verbal reasoning scores, but in contrast to S5, patient S10 also had a good visuo-spatial working memory, although it was not sufficient to perform the non-symbolic geometry test well.

Patient S12. Frontal patients were among the most impaired in the non-symbolic geometry test, except for patient S12, who performed this test with accuracies within the normative range, both before and after surgery. Their visuo-spatial working memory and non-verbal reasoning scores were within the neurotypical range, whereas their verbal reasoning and verbal working memory were impaired. This is in line with their lesion’s location, which was circumscribed to the language areas in the inferior frontal gyrus. Interestingly, the lesion did not extend to the region dorsal to BA44, which is a region selectively involved in geometrical sequence complexity independent of memory demand [46], possibly explaining why non-symbolic geometry was spared in this patient.

## 4. Discussion

The goals of this study were twofold. First, we wanted to draw neurosurgeons’ attention to two tests that they can use to measure fundamental non-verbal abilities underlying mathematical cognition, non-symbolic numerosity, and geometry perception, thereby broadening and complementing the current evaluation of mathematics-related capacities beyond simple arithmetic. To this aim, using objective and quantitative psychophysical tests, we assessed non-verbal and visuo-spatial aspects of mathematics in oncological patients via two tests usually not included in the neuropsychological battery administered before and after surgery. Our results show that performance in the two non-symbolic tests (geometry and numerosity) was not fully captured by scores in the classical neuropsychological tests. We are aware that determining whether or not these non-symbolic tests, and especially the non-symbolic geometry test, reflect the performance in other cognitive functions measured with classical neuropsychological tests; this will require measurements on a larger population of neurotypical individuals in order to evaluate their explained variance. However, the observed dissociation between performance on the non-symbolic geometry, non-verbal reasoning, and visuo-spatial working memory described in our single cases suggests that the functions tested by the non-symbolic geometry test cannot be entirely reduced to those measured with the classical neuropsychological tests. This was clear both when considering the performance on the non-symbolic geometry test across both runs (one presumably evaluating geometrical intuition, the other assessing root memory), as well as when considering performance on the runs separately.

It is perhaps not surprising that performance on the non-verbal reasoning and the non-symbolic geometry tests could be dissociated if we consider the different nature of the two tasks: the Matrix test requires individuals to identify patterns in designs displayed simultaneously and with no time limits, while in the non-symbolic geometry test, participants are asked to identify geometrical rules by keeping in mind the trajectory followed by a square. The fact that performance on the non-symbolic geometry test can be dissociated from performance on the visuo-spatial working memory test as well is perhaps more surprising. Although counterintuitive at first glance, this result might be accounted for by considering how each sequence in the non-symbolic geometry test was generated. The sequences proposed in the non-symbolic geometry test were created to constitute a non-symbolic language and, as the verbal language, it was generated to have a recursive structure. This means that from any location of the octagon, each of the other subsequent locations can be reached through the recursive application of primitives. The working memory load necessary to remember each primitive is very low and was present in most of our patients (except for S6, who indeed also failed the non-symbolic geometry test). Once an individual is equipped with this minimal working memory capacity, they can complete the sequence by recursively applying the primitive (provided they have understood and identified the recursion inherent in the non-symbolic language). This might have been the case of patient S8, who had a working memory span of only three items and yet was quite accurate in the geometry test. In line with this interpretation, a recent magneto-encephalography study using the same stimuli as those used in the current experiment suggested that the human brain exploits sequence regularities to compress long sequences in working memory [52]. Thus, it is possible that even a patient with a limited visuo-spatial working memory could succeed in the non-verbal geometry test if they have grasped the recursive rule. Perhaps more puzzling is the performance of patient S10, who could not solve the non-symbolic geometry test despite having a visuo-spatial working span within the neurotypical range. This result suggests that the non-symbolic geometry test might measure visuo-spatial working memory with higher sensitivity than the classical Corsi block tapping test. In the Corsi block tapping test, the patient can exploit 3D cues and different distances between the cubes and the edge of the board to solve the task more easily, whereas all positions are equidistant and symmetrical around the center of the screen in the non-symbolic geometry test. Future studies should compare the sensitivity of the non-symbolic geometry test with that of the Corsi block tapping test in detecting visuo-spatial working memory deficits in a larger sample of patients.

The second main goal of the study was to evaluate whether, similarly to what has been observed after acute lesions, the functional outcome of oncological lesions (as measured by the performance to neuropsychological tests) is in line with the presumed functional role of the lesioned area. We found that performance on the numerosity and non-symbolic tests was overall lower in patients with parietal and frontal lesions compared with those with occipital and temporal lesions, in line with the expected involvement of a fronto-parietal network in mathematics-related abilities. Indeed, previous neuroimaging studies have shown that perceptions of numerical quantities and geometrical reasoning recruit a wide fronto-parietal network in neurotypical individuals [33,34,46,53,54]. However, beyond this group-level result, we observed a high degree of within-group variability, with some patients with parietal and frontal lesions scoring within the neurotypical range, and others with occipital and temporal lesions scoring below it in both the non-symbolic numerosity and geometry tests. The heterogeneity in performance across patients was even more evident when considering the scores in the classical neuropsychological tests, which very poorly predicted the presumed functional role of the lesioned areas. For example, previous imaging and neuropsychological case studies identified the prefrontal cortex as a key region supporting visuo-spatial working memory during the Corsi block tapping test, or modified/computerized versions thereof (for a review, see: [55]). Based on this evidence, we expected patients with frontal lesions to show the strongest deficits in visuo-spatial working memory. At odds with this expectation, in the examined sample, only one patient with a parietal lesion was strongly impaired in the Corsi block tapping test. These findings suggest that the impact of oncological lesions on cognition might be very different compared with the impact provoked by other types of lesions, such as those related to a stroke, which typically characterize the neuropsychological literature. Tumors with a different histology have a different course and degree of invasiveness over time in the brain parenchyma and in the white matter tracts. Slowly progressing tumors leave more space for neuroplastic compensatory phenomena to occur, leading to the reorganization of the cortical structures, supporting various cognitive abilities. This factor might explain the variability in the current results. For example, in two patients, we observed largely spared numerosity discrimination abilities, despite the lesions being in the parietal cortex and including the intraparietal sulcus, a structure thought to be crucial for numerosity perception. These patients were affected by low-grade glioma (S8) and lung metastasis (S9), both characterized by slow progression rates. It is thus possible that massive plastic reorganization of cortical functionality and connectivity might have occurred in these patients, allowing them to preserve abilities that crucially rely on the lesioned structures, such as numerosity discrimination. Among those with frontal lesions, all but one patient (S12) showed impaired performance on the non-symbolic geometry test. Patient S12 was affected by glioblastoma, which is characterized by a rapid progression rate, thereby only allowing very little or no plastic reorganization, and making the lesion most similar to an acute event in its impact on cognition. In these cases, the precise location of the lesion might be more predictive of the cognitive deficit. Importantly the lesion of patient S12 was limited to the language areas (mainly BA44) and spared the more dorsal region that is specifically activated in neurotypicals during non-symbolic geometrical reasoning [46]. The neurotypical performance of patient S12 supports the key role of this region to perform the non-symbolic geometry test accurately.

Overall, the poor relation between lesion location and the neuropsychological profile observed in many patients in this study suggests that, while a certain degree of correlation between cognitive functions and cortical areas can be expected in neurotypical individuals and in neuropsychological cases due to acute events, an excessively rigid localizationist approach should be avoided when evaluating oncological patients. More generally, even if a certain function is thought to mostly rely on a set of relatively well localized regions, these are hardly the only ones supporting it; therefore, the rigid localizationist approach which often guides neurosurgeons for practical reasons is likely to fail in guaranteeing the expected outcome (i.e., saving a certain cognitive function). For example, neuroimaging evidence in healthy individuals suggests that non-symbolic abilities might rely on the functionality of other areas beyond the fronto-parietal region. Indeed, numerosity maps have been identified in multiple locations in the parietal and frontal regions, but also in temporal–occipital cortices [54,56]. Moreover, other fMRI studies have successfully decoded the numerosity of ensembles from the patterns of activity read out from the temporal and occipital cortices, in addition to the classical fronto-parietal network [53,57,58,59,60,61,62]. Interestingly, the temporal lesion in patient S2, who performed the numerosity task with poor precision, covered the superior temporal sulcus, which is one of the temporal areas from which numerosity information has been decoded in neurotypicals [59]. As for the geometry test, the only previous fMRI study that used very similar stimuli to the one used here mainly reported and discussed the activity elicited in the fronto-parietal network [46], but this being the test related to geometry, a contribution of occipito-temporal shape-sensitive regions can be expected. Overall, although the contribution of occipito-temporal areas in processing numerical and geometrical information has been under-reported in literature and its role remains unclear, claiming that these tests only evaluate the functioning of frontal and parietal regions might be oversimplistic.

Six patients were also tested after the surgery, in the post-operative period before discharge. Cognitive abilities in these patients were mostly not altered by the surgery, and in some cases even led to the recovery of lost cognitive functions, likely due to reductions in brain edema after the surgery. As we observed some improvement in cognition as soon as seven days after the surgery, even greater improvements can be expected with later follow-up.

The current study has both limitations and strengths. We wanted to select patients with small and well-localized lesions; therefore, from a sample of more than 400 neuro-oncological patients operated on at the Neurosurgical Department of the University Hospital of Florence over approximately two years, we enrolled only 16 patients, and only 6 were available for testing after the surgery. In addition, the selected patients were affected by tumors with a different histology, thus making the sample size small and very heterogeneous. Another limitation is that not all cognitive functions were directly measured (e.g., attention, cognitive control, etc.), although most of them were likely necessary to carry out the administered tests, and the subtests selected were only meant to provide a proxy measure for a given function. Considering that patients had already been tested for more than two hours, it was not conceivable to carry out a complete and detailed neuropsychological assessment. In order to quantify the independence between the cognitive abilities measured by the two psychophysical tests from those measured by classical neuropsychological tests, as well as to provide standardized norms, the current study should be replicated on a much larger population of neurotypical adults, potentially administering a wider set of neuropsychological tests. At the same time, the carefully selected pool of patients constitutes a strength of this study, in that it enabled us to embrace the single case approach, considered by many authors to be a powerful method to describe single or double dissociations between cognitive functions [51]. Moreover, testing patients, and specifically oncological rather than acute patients, gave us the unique opportunity to ascertain that anticipating specific neuropsychological deficits given the lesion location may not be always reliable, even in the presence of a small lesion.

In conclusion, the classical neuropsychological assessments could be enriched by including tests evaluating a wider range of cognitive and perceptual functions. The two non-symbolic tests measuring the perception of numerical quantities and geometrical reasoning proposed in this study could provide a more comprehensive and refined assessment of visuo-spatial and mathematical-related abilities. These psychophysical tests objectively measure cognitive and perceptual functions that are likely different from those measured in other classical neuropsychological tests, and can therefore complement them. Future studies should further optimize these tests and potentially modify the current paradigms, making them more suitable for assessment during awake surgery. Importantly, however, neurosurgeons should be aware of the fact that the neuropsychological profile of patients with oncological lesions might be poorly predicted by the lesion’s site compared with that caused by acute events. Future studies should evaluate whether the link between lesion site and cognitive impairment is more reliable, at least when considering rapidly progressive tumors only. Patients with more slowly progressing tumors might benefit more from a broader battery of tests evaluating several functions beyond those expected to be impaired simply based on the lesion location. As the testing time should be kept as short as possible to maintain a high level of compliance and be compatible with the surgery duration, one potential solution could be to develop batteries that test multiple functions at the same time, in an attempt to record all of them simultaneously.

## Figures and Tables

**Table 1:** Individual participants’ results. The table shows patients’ demographic and clinical data along with their scores for the classical neuropsychological tests, as well as performance on the two non-symbolic tests (non-symbolic numerosity discrimination and non-symbolic geometry tests). Letters correspond to the lesion main location (AG, angular gyrus; SMG, supramarginal gyrus; MFG, medial frontal gyrus; IFG, inferior frontal gyrus; SFG, superior frontal gyrus) and to tumors’ histology or pathology (A, astrocytoma; Ca, carcinoma; Co, choriocarcinoma; G, glioblastoma; M, metastasis; Avm, arteriovenous malformation; O, oligodendroglioma). Cells containing two numerical values indicate patients’ scores before (upper value) and after (lower value) the surgery. Scores lower than the normative value to the standardized neuropsychological tests or outside the 99th percentile calculated on neurotypicals in the non-symbolic tests are marked in red. n.a., not available.

Patient	Lesion Location and Size (mm)	Pathology	Age	Non-Verbal Reasoning	VSWM	Verbal Reasoning	Verbal WM	Numerosity (CV)	Geometry (Acc)	Geometry (Acc 1st Run)	Geometry (Acc 2nd Run)
S1	Right occipital lobe (23 × 17 × 20)	G	55	4	5	9	9	0.14	0.59	0.62	0.57
S2	Left temporal gyrus (20 × 20 × 20)	Avm	53	12 7	3.5 4	4 7	5 3	0.44 0.46	0.55 0.53	0.54 0.54	0.55 0.53
S3	Right middle temporal gyrus (9 × 9 × 9)	Ca	39	8 1	4 4	3 2	6 6	0.26 0.28	0.70 0.72	0.67 0.79	0.72 0.68
S4	Left temporal–parietal junction (34 × 27 × 30)	G	68	8	4.5	7	10	0.27	0.67	0.58	0.72
S5	Left AG (22 × 22 × 22)	Ca	45	n.a. 8	0 0	11 0	n.a. 6	n.a. 0.17	0.11 0.45	0.12 0.42	0.10 0.48
S6	Right parietal AG/SMG (34 × 28 × 25)	G	68	7 7	4 4.5	13 13	13 10	n.a. n.a.	0.42 0.41	0.42 0.38	0.42 0.43
S7	Right parietal (42 × 40 × 30)	G	55	5	4	3	5	n.a.	0.27	0.25	0.27
S8	Left parietal lobe (30 × 28 × 34)	O	28	5 7	6.5 5	3 4	9 1	0.2 n.a.	0.77 0.75	0.71 0.83	0.80 0.70
S9	Left parietal lobe (30 × 27 × 30)	M	56	12	4.5	8	13	0.28	0.73	0.58	0.82
S10	Right frontal lobe (MFG/IFG) (40 × 29 × 36)	G	64	10	7.5	10	n.a.	0.52	0.22	0.25	0.20
S11	Right SFG (10 × 10 × 10)	M	46	7	5	10	11	0.15	0.47	0.5	0.45
S12	Left IFG (fronto-opercular) (10 × 12 × 10)	G	54	77	5 4.5	6 5	6 5	0.18 0.29	0.77 0.73	0.79 0.75	0.75 0.73
S13	Right frontal lobe (61 × 40 × 40)	A	58	3	5	8	10	0.29	0.59	0.62	0.57
S14	Left frontal lobe (MFG/IFG) (50 × 24 × 20)	Co	29	4	4.5	1	4	0.66	0.34	0.33	0.35
S15	Left frontal lobe (SFG/MFG) (40 × 47 × 40)	G	72	6	4.5	8	10	0.49	0.41	0.38	0.42
S16	Right temporal lobe (MTG) (15 × 20 × 15)	G	67	9	5.5	12	9	0.32	0.39	0.29	0.45

## Data Availability

Data are contained within the article and Supplementary Material.

## Supplementary Materials

The following supporting information can be downloaded at: https://www.mdpi.com/article/10.3390/brainsci14010069/s1, Figure S1: Accuracy in the non-symbolic geometry test. Table S1: Clinical and anamnestic data.
