# Supplementary material for: Neurocognitive Assessment of Mathematics-Related Capacities in Neurosurgical Patients
Source: Brain Sci. 2024 Jan 10;14(1):69. doi: 10.3390/brainsci14010069 (PMC10813954; doi:10.3390/brainsci14010069)
Supplement: Supplementary file 1 [file brainsci-14-00069-s001.zip › brainsci-2805710-supplementary.pdf]

Supplementary material

Supplementary Table S1. Clinical and anamnestic data. Supplementary Table S1 reports additional clinical and anamnestic data. Antiep = antiepileptic drug.

**Patient.**	**Drug Therapy**	**Gender**	**Years of education**	**Profession**	**Social status**
S1	antiep	M	8	construction worker	married
S2	antiep	M	8	office worker	married
S3	antiep	F	8	housewife	married
S4	no	M	5	pensioner	married
S5	antiep	F	11	n.a.	married
S6	antiep	F	8	n.a.	n.a.
S7	no	F	13	n.a.	married
S8	no	M	13	businessman	not married
S9	antiep	M	11	architect	divorced
S10	antiep	M	17	doctor	married
S11	antiep	M	13	driver	married
S12	antiep	M	17	technician	married
S13	no	M	13	artisan	married
S14	antiep	M	13	n.a.	not married
S15	antiep	F	13	n.a.	married
S16	no	M	11	pensioner	married

The non-symbolic geometry test was composed of two runs, the first one presumed to rely more on geometrical intuition, the second on memory. We analyzed participants' performance on these two runs separately. Overall results to the first and second runs (Figure S1) were very homogeneous and in line with the merged results discussed in the main text, meaning that in most of the patients there was not a clear dissociation in performance between the first and second runs. Only one patient with a parietal lesion showed impaired performance in the first, but not in the second run before the surgery, and one patient with a temporal lesion showed the opposite pattern after surgery. Few cases showed only a tendency for dissociation between the first and the second runs, with scores in one of the two runs being borderline with the neurotypical range. The two runs were supposed to test sequence anticipation and memory, which could be expected to be partially measured by non-verbal reasoning and non-verbal working memory tests respectively. However, the dissociations between the first and the second runs did not clearly align the patients' scores to the classical neuropsychological tests evaluating these respective functions. A Bootstrap sign test performed on accuracies of the first run revealed significantly higher mean accuracies in patients with temporal and occipital lesions compared to both those with a frontal lesion (p=0.02) as well as compared to those with a parietal lesion (p=0.02). When the bootstrap sign test was performed on the

second run, mean accuracies in patients with temporal and occipital lesions were higher only compared to those with a frontal lesion (p=0.006).

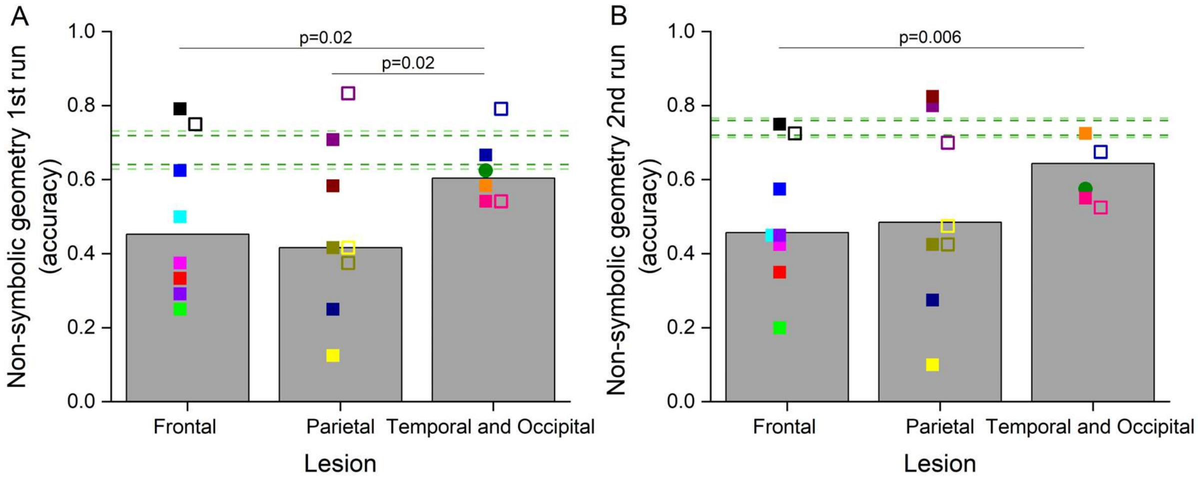

Figure S1. Accuracy in the non-symbolic geometry test. Bars represent the average results to the first (A) and the second (B) runs of the non-symbolic geometry test in the three groups of patients before the surgery. Symbols represent individual accuracies both before (filled symbols) and after (open symbols) the surgery. Color-coding is the same as the one used in Figure 2. Dashed horizontal lines represent the 95th and 99th percentile of the neurotypical scores measured in primary school children (dark and light green lines respectively).
